# Effects of rhythmic dumbbell upper limb rehabilitation training based on the multi-process action control framework on vascular access function and quality of life in hemodialysis patients

**DOI:** 10.3389/fsurg.2026.1738033

**Published:** 2026-03-02

**Authors:** Jing Hu, Mingcong Cao, Rufu Jia, Keli Pan, Xuelian Jiang

**Affiliations:** 1 Blood Purification Room, Cangzhou Central Hospital, Cangzhou, Hebei, China; 2 President's Office, Cangzhou Central Hospital, Cangzhou, Hebei, China; 3 Nursing Department, Cangzhou Central Hospital, Cangzhou, Hebei, China

**Keywords:** arteriovenous fistula, dumbbell, hemodialysis, M-PAC theory, rhythm

## Abstract

**Objective:**

To investigate the effects of rhythmic dumbbell upper limb rehabilitation exercise based on multi-process action control (M-PAC) theory on the function and quality of life of autogenous arteriovenous fistula in hemodialysis patients.

**Methods:**

A total of 72 patients who underwent autologous arteriovenous fistula (AVF) angioplasty and had stable and regular dialysis in the hemodialysis center of Cangzhou Central Hospital from January 2025 to April 2025 were selected as the research subjects, and were randomly divided into control group (*n* = 36) and intervention group (*n* = 36). Both groups were given routine rehabilitation management. The control group was given routine ball-holding rehabilitation exercise on the upper limb of the AVF side, and the intervention group was given rhythmic dumbbell rehabilitation exercise based on M-PAC theory. The intervention began 2 weeks after the operation, and the total intervention lasted for 3 months. Doppler ultrasound was used to measure the physiological maturation of AVF in both intervention and control groups, including cephalic venous blood flow, venous diameter, and skin-to-thickness distance. Clinical maturation outcomes were recorded and observed, including pump-controlled blood flow compliance rate, target values of pump-controlled blood flow, and single-needle puncture success rate. The Chinese version of the Kidney Disease Quality of Life Questionnaire (KDQOL-SFTM) was employed to evaluate patients’ quality of life before and after the intervention.

**Results:**

Prior to the intervention, there were no significant differences between the two groups in general patient data, venous diameter, skin-to-thickness ratio, and various dimensions of quality of life, indicating balanced comparability (*χ*2/|t| ≤ 1.900, *P* ≥ 0.062). Regarding cephalic venous blood flow metrics, as preoperative values were extremely low and unstable, making measurement challenging, this study only compared post-intervention cephalic venous blood flow. For pump-controlled blood flow measurements, since patients lacked access to arteriovenous fistulas (AVF) prior to intervention, this study only analyzed post-intervention pump-controlled blood flow values. Post-intervention analysis revealed that the intervention group demonstrated significantly better outcomes in all metrics compared to the control group: superior cephalic venous blood flow, venous diameter, skin-to-vessel distance (STED), pump-controlled blood flow rate, single-needle puncture success rate, and quality of life dimensions (*χ*^2^/|t| ≥ 2.574, *P* ≤ 0.012). However, no significant difference was observed in the pump-controlled blood flow rate qualification rate (*χ*^2^ = 3.486, *P* = 0.507).

**Conclusion:**

Rhythmic dumbbell upper limb rehabilitation exercise based on M-PAC theory can promote the physiological and clinical maturity of AVF and improve the quality of life of patients.

## 1 Introduction

Maintenance Hemodialysis (MHD) is the primary treatment modality for patients with end-stage renal disease (ESRD) (1). Arteriovenous Fistula (AVF), as the preferred vascular access (2), directly affects both dialysis efficacy and the quality of life of patients (3). After AVF creation, it must mature sufficiently to be used for dialysis puncture. Poor AVF maturation often leads to the inability to perform dialysis on time or inadequate dialysis, placing a significant burden on MHD patients (4, 5). Studies have shown that 28%–53% of AVFs fail to mature clinically, rendering them unable to meet the dialysis needs, necessitating effective and scientific interventions to promote AVF maturation (6). Previous studies have indicated that upper limb muscle strength and coordination, through their “pumping” effect, can optimize hemodynamic changes, improve vascular elasticity, and promote AVF maturation and stability (7). As early as 2013, Salimi F (8) suggested that the intensity of exercise after AVF surgery should gradually increase, with the use of resistance bands, dumbbells, and other equipment to assist training. MO Y and colleagues (9) attempted to use dumbbells in promoting AVF maturation exercises, which showed certain positive effects. However, in practice, patients often exhibited poor adherence due to the monotony of the exercises. Satoh M et al. (10) demonstrated that music could stimulate the desire for exercise, providing motivation and making it easier for participants to persist.

Previous studies have demonstrated that dumbbell exercises alone can positively promote the maturation of AVF, while music-assisted interventions are effective in enhancing patients’ exercise motivation and adherence. However, in clinical practice, these approaches are often implemented in isolation and lack a coherent theoretical framework to support long-term behavioral change. In addition, repetitive and monotonous exercise patterns may induce fatigue, thereby reducing patient engagement and sustainability.

Despite the demonstrated physiological benefits of exercise-based interventions, sustained engagement in postoperative AVF rehabilitation remains challenging, particularly during the early maturation period. Effective AVF exercise programs therefore require not only appropriate physical loading, but also a structured approach to support patients in translating initial intentions into consistent, long-term exercise behavior.

To address these limitations, the present study systematically integrates rhythmic music with dumbbell exercise and, for the first time, introduces the Multi-Process Action Control (M-PAC) framework as the core management strategy. The M-PAC framework focuses on the transition from intention to action and emphasizes multiple regulatory mechanisms across different behavioral phases (11). Guided by this framework, rhythmic music was employed to enhance the enjoyment and coordination of exercise, while an individualized, progressive dumbbell-loading protocol was developed. More importantly, a structured patient management and support system was established based on the reflective, regulatory, and reflexive processes of the M-PAC framework, aiming to ensure the safe, sustainable, and effective implementation of the exercise intervention. The process and results are reported as follows.

## 2 Materials and methods

### 2.1 General information

**Figure 1:**
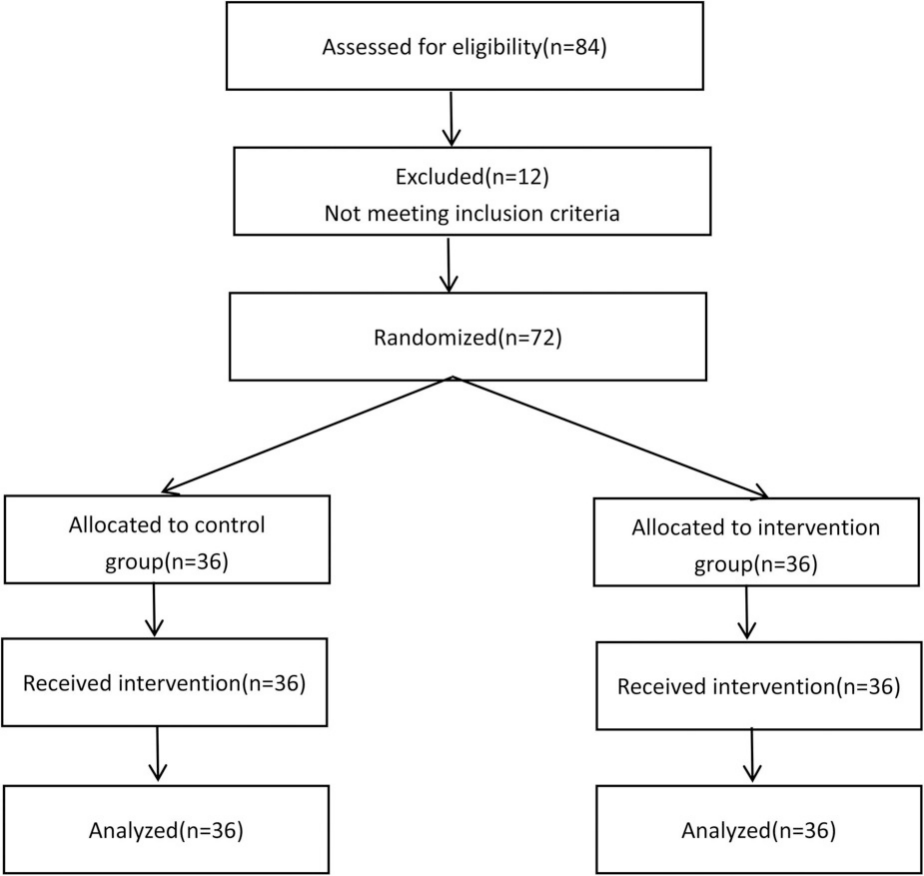
CONSORT flow diagram.

A total of 72 patients who underwent arteriovenous fistula (AVF) creation and stable regular hemodialysis at the Blood Purification Center of Cangzhou Central Hospital between January and April 2025 were selected as the study subjects. A random number sequence was generated by an external member of the research team using the Research Randomizer website (https://www.randomizer.org/), and the numbers were placed into opaque, sealed, and consecutively coded envelopes to assign the participants into a control group and an intervention group, with 36 patients in each group (Figure 1). Group assignments were concealed in sequentially numbered, opaque, sealed envelopes. These envelopes were opened only after the participants completed baseline assessments, by a research nurse who was unaware of the sequence. Baseline characteristics between the two groups were compared to ensure balance, and no significant differences were found.

The sample size was estimated based on pilot data and clinical experience. Assuming a moderate effect size (Cohen's d ≈ 0.7), a two-sided significance level (α) of 0.05, and a power of 80%, the required sample size was calculated using the formula: *n* = 2(Z_1−*α*/2_ + Z_1−*β*_)^2^/d^2^

This yielded an estimated sample size of approximately 32 participants per group. Considering a potential 10% dropout rate, the final sample size was set at 36 participants per group, totaling 72 participants.

Diagnostic Criteria: The patients met the criteria for dialysis access establishment as outlined in the “Dialysis Access China Guidelines (2024 edition)” published by the Chinese Society of Nephrology (12). Specifically, dialysis access should be planned when the estimated glomerular filtration rate (eGFR) is between 15 and 30 mL·min^−1^·(1.7 3m^2^)^−1^ or when clinical symptoms are significant and conservative treatment is insufficient.

Inclusion Criteria:

(1) Age ≥ 18 years;
(2) Surgical creation of AVF at the radial artery and cephalic vein in the forearm wrist area;
(3) First-time creation of AVF;
(4) No history of psychiatric disorders, language, or hearing impairments.

Exclusion Criteria:

(1) History of AVF creation;
(2) Presence of limb movement disorders or other issues preventing dumbbell exercises;
(3) Severe comorbid conditions, such as heart disease or malignant tumors.

Exclusion and Dropout Criteria:

(1) Development of complications such as AVF occlusion during the intervention period;
(2) Failure to complete follow-up examinations or assessments;
(3) Voluntary withdrawal by the patient.

This study has been approved by the Ethics Committee of Cangzhou Central Hospital (Ethics Approval No. 2024-262-01). All patients provided informed consent and voluntarily participated in the study.

### 2.2 Methods

Both groups received routine postoperative management following AVF creation. The control group performed grip ball exercises for rehabilitation, while the intervention group engaged in rhythmic dumbbell rehabilitation exercises based on the M-PAC (Multi-Process Action Control) theory. The total duration of the intervention was 3 months.

#### 2.2.1 Control group

Routine postoperative management was provided. Preoperatively, patients were educated on protecting the AVF side limb. Postoperatively, patients were instructed on methods to alleviate swelling and pain, as well as wound exudation and pain management. Dressings were changed every 2–3 days, and patients were informed not to have blood drawn from or rest on the AVF side limb during sleep. After suture removal, patients performed grip ball rehabilitation exercises on the surgical side arm. A medium-hardness, 7–7.5 cm diameter dialysis-specific grip ball was used. Patients were instructed to grip the ball firmly until the finger joints were maximally flexed, holding for 5 s, then completely relaxing for 5 s. This exercise was performed for 15 min per session, three times daily.

#### 2.2.2 Intervention group

(1) Personalized Dumbbell Selection: Dumbbell selection strictly followed the core principle of “avoiding heavy lifting with the AVF-side arm.” Previous research has indicated that a 6-pound weight is the threshold for red muscle fiber activation (13). For safety and effectiveness, a 6-pound dumbbell was initially selected for the first week of exercise. After one week, grip strength on the AVF side was measured weekly using a hand dynamometer. Based on the patient's dynamic grip strength, the dumbbell weight was adjusted. Referring to the study by Zhang Jialong and colleagues (14), the maximum dumbbell weight was set to no more than 30% of the patient's grip strength test result.
(2) Personalized Rhythmic Selection: Previous research (15) has indicated that the appropriate rhythm range for asynchronous music use in physical exercise is 120–140 beats per minute (bpm), with the music tempo ideally matching the rhythm of the exercise. Therefore, this study selected music with a tempo of 130 bpm, of a type that the patient found personally comfortable. The aim was to make the exercise more rhythmic, improving the patient's coordination and sense of rhythm. The exercise followed the music's rhythm: lifting during one beat and resting during the next, aligning the exercise rhythm with the music rhythm. Music Library and Selection Criteria: This study utilized a standardized music library curated by the researchers. All musical pieces had a tempo of 130 beats per minute and primarily included genres such as light popular piano music, Chinese traditional instrumental music, and light music integrated with natural sounds. Personalized Selection Process: During the intervention, patients selected the type of music they most preferred and found most comfortable. Music Delivery and Application: The music was played through the patient's own smartphone and was continuously played throughout the rhythmic dumbbell rehabilitation exercise session. The use of headphones was recommended to minimize external distraction.
(3) Exercise Method: The patient should be seated with the AVF-side upper arm naturally hanging by the side of the body, with a slight flexion at the elbow and the wrist in a neutral position. Holding the dumbbell with the palm facing the body, the movement should only occur at the shoulder joint, swinging the arm forward. The arm should be imagined as a pendulum. The initial range of motion should be small, 15–30 degrees, avoiding raising the arm above the shoulder or excessive abduction. As the patient adapts and their capability improves, the range may be gradually increased to 45 degrees, provided there is no discomfort or weakening of the AVF bruit. The maximum range should not exceed 60 degrees. Exercises should be performed three times a day for 10 min each time.
(4) M-PAC Multi-Process Action Control Exercise Management:

Reflection Process: Before the intervention, the benefits and necessity of the rehabilitation exercise were explained to the patients using simple language, addressing “Why do this?” (to protect the fistula and improve function), “What can I do?” (ability), “Is it convenient?” (opportunity), and “Can I make it a habit?” (automaticity). Clear explanations were provided on the selection of dumbbell weight and range limitations, ensuring that patients understood that this was a tailored and safe plan.

Regulation Process: The patient was involved in selecting personalized dumbbells and music rhythm, and a daily exercise plan was created. Patients were instructed to set up a convenient exercise corner at home, with a stable chair, accessible dumbbells, and a music-playing device. Family members/caregivers were encouraged to understand the plan and assist in the early stages by supervising posture, reminding the patient of exercise times, and offering positive encouragement.

Reflection Process: Monthly follow-up visits were conducted, during which feedback on the rhythmic dumbbell exercises was gathered. A physical examination and ultrasound were performed on the AVF-side arm to check for any adverse events. Patients were asked about any difficulties or challenges they encountered during the exercise process, and they were encouraged to continue their efforts.

### 2.3 Observation indicators

The following indicators were compared between the two groups before and after the intervention.

#### 2.3.1 Physiological maturation of AVF

According to the “Expert Consensus on Vascular Access for Hemodialysis in China (2nd Edition)” (16), the criteria for determining physiological maturation of AVF are: natural blood flow in the AVF puncture segment >500 mL/min, venous diameter ≥5 mm, and a skin-to-vessel distance (STED) of less than 6 mm. In this study, ultrasound measurements were used to assess blood flow in the cephalic vein 5 cm above the puncture site, as well as vessel diameter and the distance from the vessel to the skin (17).

#### 2.3.2 Clinical maturation of AVF

According to the definition of insufficient blood flow in the “Expert Consensus on Vascular Access for Hemodialysis in China (2nd Edition)” (16), if the pump-controlled blood flow during dialysis is less than 200 mL/min, the clinical maturation of the AVF is considered inadequate. This study evaluated the pump-controlled blood flow rate, the percentage of patients meeting the target pump-controlled blood flow rate, and the success rate of the first puncture within one week after the initial puncture at the end of the 3-month intervention.

Ultrasound assessments and puncture success evaluations were conducted by staff who were blinded to group allocation to minimize observer bias.

#### 2.3.3 Quality of life

The Chinese version of the Kidney Disease Quality of Life Short Form (KDQOL-SFTM) (18) was used to assess quality of life. This scale includes five dimensions: physical health, mental health, impact of kidney disease, kidney burden, and symptoms and discomfort. Each dimension is scored from 0 to 100, with higher scores indicating better quality of life. The Cronbach's α coefficient for the total scale was 0.902.

### 2.4 Statistical analysis

Statistical analysis was performed using SPSS version 26.0. Categorical data were expressed as frequencies, and the comparison between groups was conducted using the chi-square (*χ*^2^) test. The Levene test was used to assess the homogeneity of variances, and continuous data that met the normal distribution were expressed as mean ± standard deviation ($\bar{x}$ ± s). Within-group comparisons were performed using paired t-tests, while between-group comparisons were conducted using independent *t*-tests. A significance level of α = 0.05 was adopted.

## 3 Results

### 3.1 Comparison of the general conditions of patients in the two groups

The patients were randomly assigned into the control group (*n* = 36) and the intervention group (*n* = 36) using a random number table. No significant differences were observed between the two groups in terms of age, gender, stage of kidney disease, or primary etiology of the disease (*P* > 0.05). See Table 1.

**Table 1:** Comparison of baseline data between the two groups.

Group	*n*	Gender (male/female)	Age (years)	Kidney disease stage (stage 4/stage 5)	Primary etiology				Dialysis via CVC before AVF use (yes/no)	AVF side (left/right)
					Glomerulonephritis	Diabetes	Hypertension	Others		
Control group	36	21/15	55.33 ± 12.57	12/24	10	12	8	6	20/16	26/10
Intervention group	36	19/17	53.14 ± 12.07	15/21	13	10	8	5	18/18	22/14
*χ*^2^/*t*Value		0.225	0.756	0.533	0.664				0.056	0.563
*P* Value		0.635	0.452	0.465	0.882				0.813	0.453

### 3.2 Physiological maturation of AVF

Due to weak, fluctuating, or intermittent cephalic vein signals before AVF creation, it was technically difficult to obtain reliable blood flow measurements using Doppler ultrasound. Therefore, preoperative flow data were not included in the analysis. This limitation occurred at a similar frequency in both groups and is unlikely to affect baseline comparability. Before the creation, there were no significant differences between the two groups in terms of vessel diameter and distance from the skin (*P* > 0.05). After the intervention, both groups showed significant increases in cephalic vein blood flow and vessel diameter, and a significant decrease in the STED (*P* < 0.05). The changes in the intervention group were significantly higher than those in the control group (*P* < 0.05) See Table 2.

**Table 2:** Comparison of AVF physiological maturity indexes between the two groups.

Variable	Group	*n*	Test	$\bar{x}\pm$s	*T* Value	*P* Value
Cephalic vein blood flow (mL/min)	Control group	36	Post-intervention	857.33 ± 117.41		
	Intervention group	36	Post-intervention	1,214.53 ± 146.28		
	Difference between groups post-treatment			357.19 ± 199.57	11.426	<0.001
Venous diameter (mm)	Control group	36	Pre-intervention	2.25 ± 0.65	−14.962	<0.001
			Post-intervention	4.72 ± 0.69		
	Intervention group	36	Pre-intervention	2.37 ± 0.54	−22.989	<0.001
			Post-intervention	6.35 ± 0.81		
	Difference between groups pre-treatment			0.83 ± 0.53	0.892	0.376
	Difference between groups post-treatmen			1.63 ± 1.03	9.201	<0.001
STED (mm)	Control group	36	Pre-intervention	6.03 ± 1.00	8.572	0.047
			Post-intervention	4.58 ± 0.59		
	Intervention group	36	Pre-intervention	6.04 ± 0.91	10.425	<0.001
			Post-intervention	3.54 ± 0.55		
	Difference between groups pre-treatment			1.17 ± 0.84	0.055	0.956
	Difference between groups post-treatment			1.04 ± 0.30	7.725	<0.001

### 3.3 Clinical maturation of AVF

Three months after AVF creation, in the control group, 2 patients had pump-controlled blood flow rates lower than 200 mL/min, resulting in a clinical maturation rate of 94.44%. In the intervention group, all patients had pump-controlled blood flow rates greater than 200 mL/min, with a clinical maturation rate of 100%. Although the intervention group had a higher maturation rate than the control group, no statistically significant difference was found between the two groups (*P* > 0.05), as shown in Table 3. Regarding the specific values of pump-controlled blood flow, the intervention group was significantly higher than the control group (*P* < 0.05), as shown in Table 4.

**Table 3:** Comparison of pump-controlled blood flow rate and success rate of single needle puncture between the two groups.

Variable	Category	Control group (*n* = 36)	Intervention group (*n* = 36)	*χ*^2^ Value	*P* Value
Pump-controlled blood flow rate achievement	Achieved	34	36	–	0.507^a^
	Not achieved	2	0		
Puncture success rate per needle	Success	216	216	17.926	< 0.001
	Failure	51	15		

^a^ Fisher's exact test.

**Table 4:** Comparison of pump-controlled blood flow values between the two groups (mL/min).

Group	*n*	Test	$\bar{x}\pm$s	*t* Value	*P* value
Control group	36	Post-intervention	230.25 ± 17.81		
Intervention group	36	Post-intervention	257.06 ± 19.42		
Difference between groups post-treatment			29.53 ± 23.25	6.103	<0.001

After the regular AVF puncture began, all patients underwent dialysis three times a week, with at least two needles per dialysis session. The control group had a total of 267 punctures, with 51 failures and 216 successes. The intervention group had a total of 231 punctures, with 216 successes and 15 failures. The success rate of puncture per needle in the intervention group was significantly higher than that in the control group (*P* < 0.05), as shown in Table 3.

### 3.4 Quality of life

In the present study, the Cronbach's α coefficient for the overall scale was 0.92, while the coefficients for each subscale ranged from 0.76 to 0.89. Before the intervention, there were no significant differences in the quality of life between the two groups (*P* > 0.05). After the intervention, the quality of life in the control group showed no significant change, while the intervention group showed improvement. The quality of life in the intervention group was significantly better than that in the control group (*P* < 0.05). Inter-group comparisons revealed that after the intervention, patients in the intervention group scored significantly higher than those in the control group across all five domains (Physical Health: t = −3.250, *P* = 0.002; Mental Health: t = −5.584, *P* < 0.001; Impact of Kidney Disease: t = −2.574, *P* = 0.012; Kidney Burden: t = −6.953, *P* < 0.001; Symptoms and Discomfort: t = 4.173, *P* < 0.001). Specifically, the physical health score in the intervention group (73.09 ± 10.39) was approximately 7.5 points higher than that in the control group (65.58 ± 9.03), with the most pronounced advantage observed in the mental health domain (69.89 ± 10.40 vs. 57.86 ± 7.56). See Table 5.

**Table 5:** Comparison of quality of life before and after intervention between the two groups.

Variable	Group	*n*	Test	$\bar{x}\pm$s	*t-*Value	*P-*Value
Physical health (score)	Control group	36	Pre-intervention	56.36 ± 10.23	−4.480	<0.001
			Post-intervention	65.58 ± 9.03		
	Intervention group	36	Pre-intervention	55.51 ± 9.50	−7.764	<0.001
			Post-intervention	73.09 ± 10.39		
	Difference between groups pre-treatment			9.50 ± 7.65	0.361	0.719
	Difference between groups post-treatment			12.11 ± 7.77	−3.250	0.002
Mental health (score)	Control group	36	Pre-intervention	49.89 ± 9.57	−4.943	<0.001
			Post-intervention	57.86 ± 7.56		
	Intervention group	36	Pre-intervention	51.66 ± 8.88	−8.936	<0.001
			Post-intervention	69.89 ± 10.40		
	Difference between groups pre-treatment			11.81 ± 8.52	−0.807	0.423
	Difference between groups post-treatment			13.94 ± 10.63	−5.584	<0.001
Impact of kidney disease (score)	Control group	36	Pre-intervention	48.61 ± 7.30	−10.940	<0.001
			Post-intervention	68.03 ± 9.39		
	Intervention group	36	Pre-intervention	47.80 ± 5.47	−15.048	<0.001
			Post-intervention	74.06 ± 10.34		
	Difference between groups pre-treatment			5.97 ± 4.39	0.529	0.599
	Difference between groups post-treatment			12.61 ± 8.86	−2.574	0.012
Kidney burden (score)	Control group	36	Pre-intervention	52.00 ± 6.31	−8.899	<0.001
			Post-intervention	64.47 ± 6.29	−16.820	<0.001
	Intervention group	36	Pre-intervention	49.26 ± 5.84		
			Post-intervention	77.11 ± 8.85		
	Difference between groups pre-treatment			5.22 ± 3.94	1.900	0.062
	Difference between groups post-treatment			13.14 ± 4.70	−6.953	<0.001
Symptoms and discomfort (score)	Control group	36	Pre-intervention	55.17 ± 7.41	−7.037	<0.001
			Post-intervention	67.92 ± 8.77	−11.205	<0.001
	Intervention group	36	Pre-intervention	55.11 ± 8.50		
			Post-intervention	76.71 ± 9.00		
	Difference between groups pre-treatment			8.08 ± 6.67	0.028	0.978
	Difference between groups post-treatment			9.31 ± 1.92	−4.173	<0.001

95% Confidence Intervals for post-treatment differences between groups are as follows: Physical Health: 95% *CI* = [9.57, 14.65]; Mental Health: 95% *CI* = [10.47, 17.41]; Impact of Kidney Disease: 95% *CI* = [9.72, 15.50]; Kidney Burden: 95% *CI* = [11.60, 14.68]; Symptoms and Discomfort: 95% *CI* = [8.68, 9.94].

## 4 Discussion

Arteriovenous fistula (AVF) has long been considered the lifeline for patients on maintenance hemodialysis (MHD), with its proper function playing a critical role in ensuring adequate dialysis for these patients (19–21). The Dialysis Access China Guidelines (2024) (12) recommend that post-AVF creation rehabilitation exercises contribute to the stable maturation of the fistula. The European Society for Vascular Surgery (2018) (22) and the European Renal Best Practice (2019) (23) guidelines similarly emphasize adequate vessel diameter, sufficient blood flow, and superficial vein depth as key determinants of functional AVF maturation and successful cannulation. However, there is no consensus on the specific types of exercises and management methods for such rehabilitation (24–26). In this study, we made exploratory improvements based on routine AVF rehabilitation exercises by combining rhythmic music with dumbbell exercises as an intervention, and applied the M-PAC theory to ensure the continuous and effective implementation of the exercises.

The results of this study indicate that rhythmic dumbbell rehabilitation exercises based on the M-PAC theory can promote the maturation of AVF. The “China Dialysis Access Expert Consensus (2nd Edition)” (16) states that after AVF creation, puncture cannot be immediately performed, and a maturation period of 4–12 weeks is required. During this period, vascular reconstruction occurs, blood flow increases, and the vessel wall thickens and becomes more superficial. For physiological maturation, the natural blood flow in the puncture segment must be greater than 500 mL/min, the venous diameter should be ≥5 mm, and the depth from the skin must be less than 6 mm. However, physiological maturation does not necessarily equate to clinical usability. Clinical maturation is defined as achieving a pump-controlled blood flow rate of 200 mL/min or higher after the patient begins dialysis, and should also be considered in conjunction with the actual puncture conditions.

In this study, rhythmic dumbbell rehabilitation exercises, under the management of the M-PAC theory, were applied during the AVF maturation period, leading to significant increases in cephalic vein blood flow and venous diameter, as well as a reduction in the depth from the skin. These physiological maturation indicators showed notable improvement. Although the pump-controlled blood flow rate achievement did not show significant differences, the actual pump-controlled blood flow values in clinical use significantly improved, and the success rate of puncture per needle also showed significant improvement. These clinical maturation indicators suggest that rhythmic dumbbell rehabilitation exercises based on the M-PAC theory have clear effects.

Previous studies have attempted various methods of functional exercise to promote AVF maturation (27–29), including some research involving dumbbell exercises (30–32). However, these studies often lacked theoretical support and struggled with effective management in clinical practice. Additionally, the monotonous nature of the exercises led to increased patient fatigue and poor adherence. In contrast, this study utilized the M-PAC multi-process control theory as the management basis, providing stage-specific and targeted interventions for patients, ensuring the effectiveness of the rehabilitation exercises. Furthermore, the individualized rhythmic music enhanced the appeal of dumbbell exercises, making them more acceptable to patients. The selection of dumbbell weight based on grip strength ensured the safety and effectiveness of the exercises, thereby promoting AVF maturation.

The results of this study also demonstrate that rhythmic dumbbell rehabilitation exercises based on the M-PAC theory can effectively improve the quality of life in AVF patients. Prior to AVF creation, patients were troubled by disease symptoms and psychological anxiety, leading to a significant decline in quality of life. After AVF creation, with a clear treatment path and better control of disease symptoms, patients experienced some improvement in quality of life. Zhao Zhongqiu et al. (33) found that music-based exercise could improve patients’ anxiety and depression, which is consistent with the results of this study. Rhythmic dumbbell exercises improved patients’ psychological status to some extent, positively influencing their quality of life. Furthermore, the M-PAC theory management process allowed patients to experience a rigorous and scientific hospital management process, which also had a positive effect on improving their quality of life.

In this study, the pump-controlled blood flow rate did not show a significant difference (*P* > 0.05). Several factors may explain this result: First, the primary outcome in this study was blood flow measured by ultrasound. The sample size calculated for a “rate” outcome based on this data was relatively small, which could have led to a false negative result due to insufficient sample size. Second, previous literature (34) has pointed out that when AVFs experience complications such as stenosis or thrombosis, ultrasound shows significant changes in blood flow, but the pump-controlled blood flow and dialysis adequacy may not immediately reflect these changes. This suggests that pump-controlled blood flow can only serve as an auxiliary reference when monitoring AVF function, and ultrasound results may provide a more timely and reliable assessment.

## 5 Conclusion

Rhythmic dumbbell exercises based on the M-PAC theory are beneficial for promoting the physiological and clinical maturation of AVF in MHD patients, as well as improving their quality of life. These exercises have potential for wider application.

## 6 Limitation

There are certain limitations in this study. This was a single-center study with an insufficient sample size. Additionally, the follow-up period could be extended to further investigate the impact of this rehabilitation exercise on long-term complications related to AVF. We acknowledge that several potential confounding factors—such as blood pressure, diabetes, vascular calcification, surgeon variability, antiplatelet use, and needling technique—may influence AVF maturation and puncture outcomes. However, these variables were not comprehensively collected or adjusted for in this study. This represents a limitation and may affect the interpretation of the intervention effect. Future studies should consider stratifying or adjusting for these confounders to ensure more accurate assessment of causality.

## Data Availability

The datasets presented in this article are not readily available because the dataset generated and analyzed during the current study is available only to members of the research group and is not publicly accessible. Requests to access the datasets should be directed to 1198138073@qq.com.

## References

[1] Alghamdi LS Alonazi W. The utilization of renal dialysis: a comprehensive study in Saudi Arabia. BMC Public Health. (2024) 24(1):1914. 10.1186/s12889-024-19450-5 39014360 PMC11253410

[2] Lee J Lee S Chang JW Kim SW Song JK. Clinical value of intraoperative flow measurements of brachiocephalic arteriovenous fistulas for hemodialysis. Korean J Thorac Cardiovasc Surg. (2020) 53(3):121–6. 10.5090/kjtcs.2020.53.3.121 32551292 PMC7287223

[3] Lok CE Huber TS Lee T Shenoy S Yevzlin AS Abreo K KDOQI clinical practice guideline for vascular access: 2019 update. Am J Kidney Dis. (2020) 75(4 Suppl 2):S1–S164. 10.1053/j.ajkd.2021.02.002 32778223

[4] Northrup H He Y Berceli S Cheung AK Shiu YT. Arteriovenous fistula histology, hemodynamics, and wall mechanics: a case report of successful and failed access in a single patient. Kidney Med. (2024) 6(4):100801. 10.1016/j.xkme.2024.100801 38562969 PMC10982562

[5] Wang P Chen H. Research progress on the factors influencing poor maturation of autologous arteriovenous fistula. Chin J Nephrol. (2023) 23(7):585–9. 10.3969/j.issn.1671-2390.2023.07.009

[6] Nousis A Tziastoudi M Oustampasidou N Efthymiadi M Divani M Eleftheriadis T Epidemiology of vascular access in patients undergoing chronic hemodialysis treatment in Greece. J Clin Med. (2025) 14(13):4571. 10.3390/jcm14134571 40648941 PMC12249874

[7] Khattri RB Kim K Anderson EM Fazzone B Harland KC Hu Q Metabolomic profiling reveals muscle metabolic changes following iliac arteriovenous fistula creation in mice. Am J Physiol Renal Physiol. (2022) 323(5):F577–89. 10.1152/ajprenal.00156.2022 36007889 PMC9602894

[8] Salimi F Majd Nassiri G Moradi M Keshavarzian A Farajzadegan Z Saleki M Assessment of effects of upper extremity exercise with arm tourniquet on maturity of arteriovenous fistula in hemodialysis patients. J Vasc Access. (2013) 14(3):239–44. 10.5301/jva.5000123 23283645

[9] Mo Y Song L Sun C Huang J Zhou L Zheng S Effect of dumbbell exercise on arteriovenous fistula in patients undergoing maintenance hemodialysis: a prospective randomized controlled trial. Blood Purif. (2020) 49(1–2):16–24. 10.1159/000502332 31536984

[10] Satoh M Ogawa J Tokita T Nakaguchi N Nakao K Kida H The effects of physical exercise with music on cognitive function of elderly people: Mihama-Kiho project. PLoS One. (2014) 9(4):e95230. 10.1371/journal.pone.0095230 24769624 PMC4000225

[11] Ting Cheung DS Chan CK Rhodes RE Chau PH Chiang CL Tse M Formative evaluation of a mobile chat-based intervention (ChatEx) for promoting exercise behaviour in older cancer survivors. Eur J Oncol Nurs. (2025) 74:102774. 10.1016/j.ejon.2024.102774 39798519

[12] Hemodiafiltration Guideline Working Group, Chinese Nephrologist Association. Clinical practice guideline for quality control of hemodiafiltration. Zhonghua Yi Xue Za Zhi. (2024) 104(8):571–93. 10.3760/cma.j.cn112137-20231212-01361 38389236

[13] Marcucci L Reggiani C Natali AN Pavan PG. From single muscle fiber to whole muscle mechanics: a finite element model of a muscle bundle with fast and slow fibers. Biomech Model Mechanobiol. (2017) 16(6):1833–43. 10.1007/s10237-017-0922-6 28584973

[14] Zhang JL Wang YH Chen JG Zhu Z Guo JH. The effect of upper limb resistance exercise on autologous arteriovenous fistula in elderly patients undergoing maintenance hemodialysis. Chin J Gerontol. (2025) 45(16):3915–9. Available online at: https://publish.cnki.net/journal/portal/zlxz/client/paper/0091d7519627b439666e024bb886cb4f

[15] Zhou Z. Research progress, model construction, and strategy suggestions for promoting physical exercise with music. J Henan Univ Educ. (2022) 31(4):58–64. 10.3969/j.issn.1007-0834.2022.04.011

[16] Chinese Hospital Association Blood Purification Center Vascular Access Working Group. Expert consensus on vascular access for hemodialysis in China (2nd edition). Chin Blood Purif. (2019) 18(6):365–81. 10.3969/j.issn.1671-4091.2019.06.001

[17] Cao MC Jia RF Wang YF Pan KL Hu J. The effects of health education and exercise style changes on the maturation of autologous arteriovenous fistula in hemodialysis patients: a randomized controlled trial. J Vasc Access. (2025) 26(1):271–9. 10.1177/11297298231214572 38053247

[18] Xu M Xu X Shi W Zhang J. Application of the Chinese version of the kidney disease quality of life short form in end-stage renal disease patients undergoing hemodialysis. Jiangsu Med J. (2015) 41(15):1814–6. Available online at: https://med.wanfangdata.com.cn/Paper/Detail?id=PeriodicalPaper_jsyy201515028&dbid=WF_QK

[19] Gatta G Scarlatella A Aucella F Nardella M Perpetuini D Tritto M Clinical thermography for the management of hemodialysis vascular access. G Ital Nefrol. (2024) 41(4):2024–VOL4. 10.69097/41-04-2024-10 39243415

[20] Zhang PY Liang JQ Wang YH Yang HJ Zhang WL Zhu L A nationwide cross-sectional survey of the use of arteriovenous fistula for hemodialysis in 30 provinces and cities in China. Chin Blood Purif. (2025) 24(6):518–23. 10.3969/j.issn.1671-4091.2025.06.016

[21] Liu F Xiang Y Liu X. Vascular access use and complications in maintenance hemodialysis patients. Chin J Hosp Infect. (2025) 35(10):1549–52. 10.11816/cn.ni.2025-241369

[22] Schmidli J Widmer MK Basile C de Donato G Gallieni M Gibbons CP Editor’s choice - vascular access: 2018 clinical practice guidelines of the European society for vascular surgery (ESVS). Eur J Vasc Endovasc Surg. (2018) 55(6):757–818. 10.1016/j.ejvs.2018.02.001 29730128

[23] Gallieni M Hollenbeck M Inston N Kumwenda M Powell S Tordoir J Clinical practice guideline on peri- and postoperative care of arteriovenous fistulas and grafts for haemodialysis in adults. Nephrol Dial Transplant. (2019) 34(Suppl 2):ii1–ii42. 10.1093/ndt/gfz072 31192372

[24] Nantakool S Reanpang T Prasannarong M Pongtam S Rerkasem K. Upper limb exercise for arteriovenous fistula maturation in people requiring permanent hemodialysis access. Cochrane Database Syst Rev. (2022) 10(10):CD013327. 10.1002/14651858.CD013327 36184076 PMC9527110

[25] He Y Northrup H Roy-Chaudhury P Cheung AK Berceli SA Shiu YT. Analyses of hemodialysis arteriovenous fistula geometric configuration and its associations with maturation and reintervention. J Vasc Surg. (2021) 73(5):1778–1786.e1. 10.1016/j.jvs.2020.09.033 33091518 PMC8055729

[26] Wilschut ED Rotmans JI Bos EJ van Zoest D Eefting D Hamming JF Supervised preoperative forearm exercise to increase blood vessel diameter in patients requiring an arteriovenous access for hemodialysis: rationale and design of the PINCH trial. J Vasc Access. (2018) 19(1):84–8. 10.5301/jva.5000826 29148008

[27] Li SY Lu Y Liu J Zhang MB Gong HJ Li MY Application of quantified grip strength training in patients after autologous arteriovenous fistula surgery. Chongqing Med. (2024) 53(11):1675–8. 10.3969/j.issn.1671-8348.2024.11.015

[28] Qiu Z. The Effect of Quantified Grip Strength Training Intensity and Frequency on the Maturation of Autologous Arteriovenous fistula. Fuzhou City: Fujian Medical University (2023). Available online at: https://med.wanfangdata.com.cn/Paper/Detail?id=DegreePaper_D03432304&dbid=WF_XW

[29] Ma J Zhang Z Tong H Zhu B Yang R Wang LS. Personalized arm restraint exercise during the maturation period after arteriovenous fistula surgery. Nurs J. (2021) 36(23):29–31. 10.3870/j.issn.1001-4152.2021.23.029

[30] Hsiao YH Shin ML Huang CP Chen SJ Huang TY. Using interdisciplinary cooperation to improve the rate of proper performance of hand exercise among hemodialysis patients with arteriovenous fistula construction. Hu Li Za Zhi. (2017) 64(3):74–81. 10.6224/JN.000042 28580561

[31] Mo Y. Randomized Controlled Study of Dumbbell Weight-bearing arm-swing Exercise on Arteriovenous fistula in Hemodialysis Patients. Guangzhou City: Southern Medical University (2020). Available online at: https://med.wanfangdata.com.cn/Paper/Detail?id=DegreePaper_Y3745104&dbid=WF_XW

[32] Feng Q Bu H Zhang L. Application of dumbbell weight-bearing arm-swing exercise in promoting arteriovenous fistula maturation in hemodialysis patients. Xinjiang Med. (2021) 51(8):866–8. Available online at: https://med.wanfangdata.com.cn/Paper/Detail?id=PeriodicalPaper_xjyx202108002&dbid=WF_QK

[33] Zhao ZQ Liang JZ Tang XQ. Research progress on the treatment of depression using the five-tone method in traditional Chinese medicine. Shaanxi J TCM. (2020) 41(3):406–8. 10.3969/j.issn.1000-7369.2020.03.038

[34] Zhao PN Ruan L Li W Xu YK Niu ZL Yang YL Study on the relationship between blood pressure, pump-controlled blood flow, and fistula flow in hemodialysis patients. Chin Blood Purif. (2023) 22(8):629–32. 10.3969/j.issn.1671-4091.2023.08.014

